# Precocious development of self-awareness in dolphins

**DOI:** 10.1371/journal.pone.0189813

**Published:** 2018-01-10

**Authors:** Rachel Morrison, Diana Reiss

**Affiliations:** 1 Department of Psychology, The Graduate Center of The City University of New York, New York, NY, United States of America; 2 Department of Psychology, Hunter College of The City University of New York, New York, NY, United States of America; Institute of Deep-sea Science and Engineering, Chinese Academy of Sciences, CHINA

## Abstract

Mirror-self recognition (MSR) is a behavioral indicator of self-awareness in young children and only a few other species, including the great apes, dolphins, elephants and magpies. The emergence of self-awareness in children typically occurs during the second year and has been correlated with sensorimotor development and growing social and self-awareness. Comparative studies of MSR in chimpanzees report that the onset of this ability occurs between 2 years 4 months and 3 years 9 months of age. Studies of wild and captive bottlenose dolphins (*Tursiops truncatus*) have reported precocious sensorimotor and social awareness during the first weeks of life, but no comparative MSR research has been conducted with this species. We exposed two young bottlenose dolphins to an underwater mirror and analyzed video recordings of their behavioral responses over a 3-year period. Here we report that both dolphins exhibited MSR, indicated by self-directed behavior at the mirror, at ages earlier than generally reported for children and at ages much earlier than reported for chimpanzees. The early onset of MSR in young dolphins occurs in parallel with their advanced sensorimotor development, complex and reciprocal social interactions, and growing social awareness. Both dolphins passed subsequent mark tests at ages comparable with children. Thus, our findings indicate that dolphins exhibit self-awareness at a mirror at a younger age than previously reported for children or other species tested.

## Introduction

Tests of mirror self-recognition (MSR) have been central to our understanding of self-awareness from developmental and evolutionary perspectives. MSR is an empirical index of emerging self-awareness in humans and this capacity has been reported to spontaneously emerge (in the absence of explicit training) in only a handful of other species including great apes [1–6], bottlenose dolphins (*Tursiops truncatus*) [7], Asian elephants (*Elephas maximus*) [8], and magpies (*Pica pica*) [9]. The phylogenetic distance between the species demonstrating MSR has been proposed as a case for cognitive convergence [7,8].

The general paradigm used to evaluate the capacity for MSR in humans and other animals involves documenting the behavioral responses of individuals during mirror exposure. Striking behavioral similarities have been reported across species showing MSR. Individuals generally progress through three basic stages: 1) mirror exploration or social behavior, 2) contingency-testing (i.e., performance of unusual & repetitive behaviors at the mirror affording the opportunity to perceive a one-to-one correspondence between the individual’s behavior & the mirror image), and 3) self-directed behavior (i.e., viewing body parts/behaviors unobservable in the mirror’s absence) [2,7,8,10,11]. In prior MSR studies, self-directed behavior has been considered a critical indicator of MSR [12] and the mark test [2], or rouge test with children [11], has been implemented after individuals exhibit self-directed behavior to further confirm this ability. During the mark test, an individual is marked on an area of the face or body that is only visible to them in a mirror. The standard criteria for passing the mark test requires that after being marked, an individual then touches, investigates, or in the case of the non-handed dolphin, orients the marked area to the mirror.

Developmental studies with children have reported that MSR, as evidenced by self-directed behavior, first emerges between 12–15 months and mark-directed behavior (passing the mark test) emerges between 18–24 months [10,11,13]. The emergence of MSR in children coincides with the onset of other indices of self and social awareness (e.g. pretend play [14] empathy and prosocial behavior [1,15,16], synchronic imitation [14,15] and may be linked to the development of sensorimotor intelligence [4,10]. Theories regarding the developing process of self-awareness in children have also proposed proprioceptive awareness as a landmark antecedent for the perception of self in a mirror [17,18]. Comparative studies with chimpanzees, report the earliest age for self-directed behavior from 24 months [13,19] to 3 years 3 months [4] and for mark-directed behavior from 2 years 4 months [13,19] to 3 years 9 months [4] (age discrepancies may be due to different criteria for self-directed behavior and passing the mark test [4,13,19]).

MSR was previously demonstrated in two adult male dolphins [7] but no comparative developmental studies of MSR have been conducted with young dolphins. Studies of wild and captive bottlenose dolphins have reported precocious sensorimotor and social development in this species. Within the first postpartum weeks, dolphin calves show advanced sensorimotor and muscle development as well as complex and reciprocal social interactions [20–26]. Studies have reported precocious locomotor muscle development in newborn calves similar to that of adult dolphins [20] and it has been reported that by one month of age, dolphins show early development of proprioception leading to advanced motor coordination [21]. Social awareness in calves increases in the postpartum weeks as they synchronize their swimming and breathing with their mother’s and recognize and reunite with them after separations [23]. Compared to infant human and nonhuman primates, dolphin calves engage with other members of their social group at a younger age. By the end of the first postpartum week, calves in the wild engage in social play behaviors with individuals other than their mothers [23] and observations conducted from birth through the 4^th^ year report that the number of associations with different individuals peaks during the first year of development [24]. The early development of social and other cognitive skills in dolphin calves, as in the case for humans, may contribute to the ability for MSR. Cognitive research conducted with young captive dolphins demonstrated that by the end of their first year they show a proclivity for spontaneous vocal and associative learning [26], skills that may contribute to the awareness of self and others.

Given the comparatively precocious development of social awareness and sensorimotor skills observed in dolphins and the reported relationship between these domains and the development of MSR in humans, we investigated whether the emergence of MSR in dolphins would develop at a comparatively young age. We exposed two young bottlenose dolphins within their social group to a mirror during a 3-year longitudinal study. We followed a similar methodology for mirror exposure as employed by Reiss and Marino [7] in their study on MSR in dolphins.

## Methods

### Subjects and procedures

Two captive born young bottlenose dolphins, Bayley (female, age 3.5 months) and Foster (male, age 14 months), were exposed to a one-way mirror with their social group of seven other bottlenose dolphins housed at the National Aquarium in Baltimore, MD. Sessions were conducted during a period from November 21, 2008 through December 15, 2011 for ~1 hour 1–2 days biweekly at ~9 am, prior to the dolphin area being open to the public. On occasion, due to scheduling issues, some sessions were conducted in the late morning, were less than 1 hour in duration, or were not conducted. Each dolphin’s behavior was videotaped in all three conditions: baseline (mirror absent), mirror (mirror present) or control (a one inch wide non-reflective frame affixed to inside of window). Baseline and control sessions were interspersed between experimental/mirror sessions. All sessions were conducted from an underwater viewing window on the wall of a ~4 ft. in diameter circular observation room that was accessible from above via a horizontal ladder and could be completely enclosed and darkened by closing a hatch door. The observation room is located between 3 interconnected pools (EP, the main exhibit pool; HP1 & HP2, two holding pools). Within the observation room, there are three windows, one into each of the pools. The observation room and the HP1 & HP2 holding pools can be accessed only by staff and research personnel. During experimental sessions, the dolphins were exposed to and tested with the mirror when they were in HP1. The number of dolphins present in the pool displaying the mirror during experimental sessions varied over the course of the study due to husbandry decisions based on the social dynamics. Thus, the dolphins were not always exposed to the mirror within the same session or with the same members of their social group. The dolphins were not housed alone and therefore individuals were not exposed to the mirror when alone.

These dolphins were not considered to be mirror naïve at the onset of the study because they had prior exposure to potentially semi-reflective windows on the walls of their pools. The main dolphin pool (EP) has large expanses of windows around the perimeter and the HP1 and HP2 pools each had one smaller observation window as previously described. The dolphin exhibit is illuminated from above by windows; the natural daylight creates differential and changing light levels that can result in the windows having mirror-like reflectivity.

### Mirror sessions

We affixed a one-way mirror (23˝ W x 35˝ H) to the outside of an underwater viewing window, which presented a reflective mirror surface to the dolphin side of the pool and a transparent surface as seen from the inside of the observation area. This enabled us to videotape all of the dolphins’ responses to the mirror. To create an optimal one-way mirror, it was necessary to completely darken the non-mirror side in the observation room by covering the remaining two windows with dark velour curtains. The authors and a member of the dolphin care staff observed from seated positions in the observation room and took notes on the identity of the dolphins at the mirror, the time of their arrival at the mirror, and their subsequent behavior. Neither the camera nor the researchers were visible to the dolphins. The researchers were silent during the sessions.

### Mark test sessions

Following the paradigm used in a previous study reporting MSR in adult dolphins [7], multiple mark tests were conducted with each dolphin after self-directed behaviors were well-documented. During mark test sessions, the dolphins were exposed to the mirror for approximately 15-minutes. The researchers then notified the trainers to approach the pool and signal the dolphins to station at the opposite side of the pool from the mirror. Using either a black ink non-toxic temporary marker (Entre Marker, Westborough, MA) or black lipstick (NK, with Vitamin E, No 306, 014729F10), a single dolphin was marked with a triangular or X-shaped mark (area ~6. 4 cm) by their trainers on various parts of their body that they could not see in the absence of the mirror. Specifically, the dolphins were marked on either side of their head behind their eye, on the palmar side of one of their pectoral fins, or on their ventral surface between their pectoral fins. After the mark was applied, the marked dolphin and other dolphins were released from station and video recordings continued to document the dolphins’ behaviors at the mirror. During these sessions, the researchers in the observation room documented the time of stationing (when the dolphin was marked), the time of release from stationing, the time of the marked dolphin’s first arrival at the mirror in the post-mark condition, and their subsequent behavior at the mirror. Due to husbandry concerns, the initial mark tests with both dolphins were delayed until they were at least 2 years old (Foster: 2 years, 2 months; Bayley: 2 years, 7 months). Also, to minimize the marking and handling of these young animals, we did not conduct sham marks prior to marking as described in Reiss and Marino [7].

### Baseline sessions

Baseline sessions were conducted to determine the dolphins’ behaviors at the window in the absence of the mirror. These sessions were videotaped in the same manner as the experimental sessions, however, in order to minimize the reflective properties of the window itself, the interior of the observation room was not darkened; in baseline conditions the windows remained uncovered and the hatch above was open. Because the dolphins could see into the observation room through the window, the video camera was started and the researchers immediately left the area until the termination of the session.

### Control sessions

To control for the placement of a novel object (the mirror) onto the window, a non-reflective clear surface (Plexiglas) was affixed to the window and video recordings were conducted. After 3 sessions, it was determined that the Plexiglas itself was reflective when mounted against the window surface and we discontinued its use. Instead, we used a 1-inch-wide non-reflective matt black poster board frame with the same dimensions as the window for the control in the remaining control sessions. The protocol used for the baseline sessions was repeated in the control sessions.

### Behavioral coding

Prior to the beginning of the study, to determine the field of visibility at the mirror from the dolphins’ point of view, a diver wearing an underwater communication device positioned himself in front of the mirror, while the dolphins were held in adjacent pools. The diver’s reflection was videotaped from the observation room as he swam up to the mirror and oriented from various positions and distances relative to the mirror. Simultaneously, the diver informed the researchers in the observation room when he could and could not view different parts of his body in the mirror.

For each dolphin, all sessions from year 1 and 1–2 mirror sessions per month for years 2 and 3 were analyzed. The dolphins’ behaviors were analyzed using an ethogram, which was based on a previous ethogram developed by Reiss and Marino [7], but expanded to include other behaviors, as well as specific codes for the dolphins’ body orientations and their distances relative to the mirror (S1 Table). In cases in which the dolphins exhibited a combination of behaviors at the same time (e.g. bubble production with inverted posture while moving head to the right and left), such composite behaviors were coded as one event. Using this system there was a high level of agreement between the observers; however, in cases in which there was a disparity in coding, the observers reviewed the video together and recoded based on their agreement. Both authors independently reviewed and coded all mark test sessions and a subset of the sessions during the first year and one author (RM) analyzed the remainder of the sessions. Post independent analysis of the mark test sessions, the two observers discussed their findings and determined if they agreed. For each mark test session, we compared the dolphin’s behavior in the pre-mark and post-mark condition to confirm that changes in the dolphin’s behavior were in response to the presence of the mark. The dolphins passed the mark test if they oriented the marked part of their body toward the mirror more frequently in the post-mark versus the pre-mark condition.

The frequency of occurrence of specific behaviors exhibited by each dolphin were quantified and categorized as either *exploratory/social behavior*, *contingency-testing*, *self-directed*, *stationing*, *or ambiguous*. *Stationing* referred to the dolphin facing the mirror for > 3 seconds, but not engaged in a specific behavior. Some behaviors were difficult to classify; to be conservative, these behaviors (e.g. approach behaviors, rostrum contact with the mirror) were classified as *ambiguous*. In early mirror sessions, repetitive behaviors first emerged and were typically classified as contingency-testing. In later sessions dolphins produced bouts of repetitive behaviors in contexts when they were reliably exhibiting self-directed behaviors thus these repetitive behaviors may be self-directed; however, to be conservative, all repetitive behaviors were categorized as contingency-testing. We also quantified the categories of behavior and the total time spent at the window in the baseline, mirror, and control conditions.

### Ethics statement

This study was approved by Hunter College’s Institutional Animal Care and Use Committee (IACUC) (Protocol #: DR dolphin MSR 1/13-02 Developmental aspects of MSR in dolphins) and the Animal Care Committee at the National Aquarium in Baltimore, MD.

## Results

A total of 57 sessions were analyzed (baseline *n* = 7, mirror *n* = 44, & control *n* = 6) (S2 Table). The dolphins produced similar behaviors described in prior MSR studies with humans [10,11,13], chimpanzees [2,4,13,27], and dolphins [7,28,29]. Throughout the study the predominant behavior exhibited at the mirror by both dolphins was self-directed (Table 1).

**Table 1:** Frequency of categorized behaviors in the mirror, baseline, and control conditions.

Category	Bayley			Foster		
	Condition					
	Mirror	Baseline	Control	Mirror	Baseline	Control
Ambiguous	242	0	8	789	6	11
Stationing	201	2	2	406	1	5
Self-directed	**356**	1	3	**1926**	1	14
Contingency-testing	90	0	1	664	2	6
Social	8	0	0	388	0	1

Numbers represent the frequency of each category of behavior for each of the conditions.

Bayley exhibited only 8 (0.9%) instances of social behavior; most of her behavior was categorized as self-directed (*f* = 356, 39.7%) (S1 Video), followed by ambiguous (*f* = 242, 27%), stationing (*f* = 201, 22.4%), and contingency-testing (*f* = 90, 10%). Foster exhibited little social behavior (*f* = 388, 9.3%); most of his behavior was categorized as self-directed (*f* = 1926, 46.2%) (S2 Video) followed by ambiguous (*f* = 789, 18.9%), contingency-testing (*f* = 664, 16%), and stationing (*f* = 406, 9.7%)

**Fig 1:**
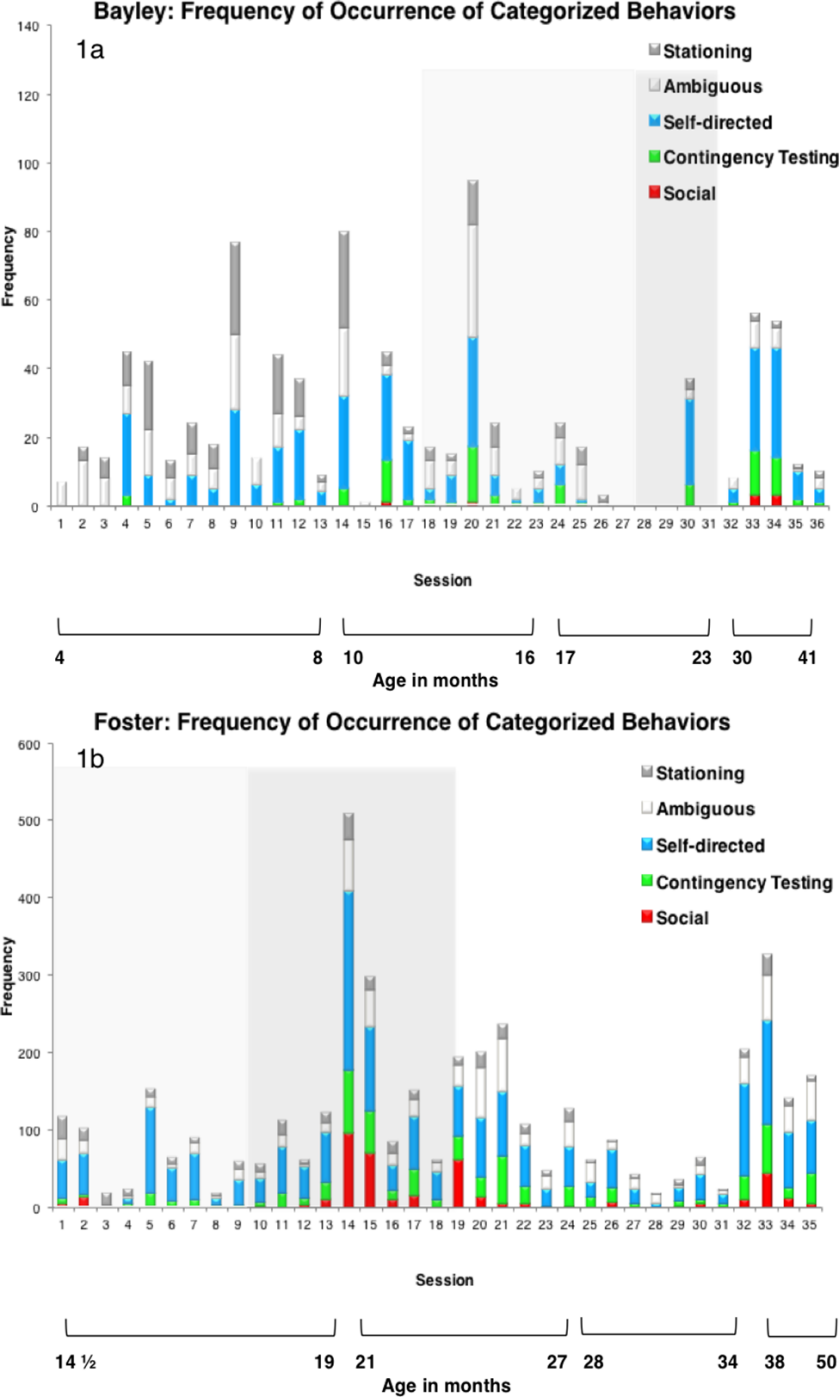
Frequency of occurrence of categorized behaviors during each day of mirror exposure for each dolphin. The shaded sections represent the age when children first demonstrate self-directed behavior (light gray, 12–15 months) and mark-directed behavior (dark gray, 18–24 months). **a**, Although Bayley’s first instances of self-directed behavior at the mirror were observed at 5.5 months old (session 4), by 7 months old (session 9) her self-directed behavior was well established. **b**, Foster exhibited predominantly self-directed behavior on his first day of mirror exposure when he was 14.5 months.

Social behavior directed toward the mirror was rarely exhibited by either dolphin, even from the onset of mirror exposure. The low incidence of social and contingency-testing behaviors at the onset of the study may be due to the dolphins’ prior exposure to the semi-reflective properties of surfaces in their pools. On the first day of mirror exposure, Bayley (age 3.5 months) spent little time at the mirror (46 sec); the few behaviors she exhibited (e.g. distant stationing, circling, & swimming by the mirror) were categorized as ambiguous (*f* = 7, 100%). No social behaviors were observed on days 1, 2 or 3 (Fig 1A). On Bayley’s 4^th^ day of mirror exposure (age 5.5 months), we first observed instances of self-directed behavior. Although she spent only a few minutes at the mirror, when there, she exhibited predominantly self-directed behavior (*f* = 24, 53.3%) (e.g. close-eye viewing, bubble production in the absence of vocalization, body tilting) and few contingency-testing behaviors (*f* = 3, 6.7%). In the following session, Bayley (age 6 months) continued to exhibit self-directed behavior in the absence of contingency-testing. By age 7 months, Bayley’s performance of self-directed behavior was well-established at the mirror. During Foster’s first day of mirror exposure (age 14.5 months), he exhibited predominantly self-directed behavior (*f* = 48, 40.7%) marked by close eye viewing, open mouth viewing, and bubble production (in the absence of vocalization). Contingency-testing was also observed, but to a lesser degree (*f* = 8, 6.8%); little social behavior (2 instances of whistle bubble streaming and 2 instances of echolocation) was observed (*f* = 4, 3.4%) (Fig 1B).

We quantified the categories of behavior and the total time spent at the window in the mirror, baseline, and control conditions. The dolphins produced more behaviors at the window during the mirror condition than during the baseline and control conditions (Table 1). We also compared the median duration of time (in seconds) each dolphin spent at the window in the different conditions (Mann-Whitney U, Bonferroni corrected alpha = 0.017). Both dolphins spent significantly more time at the window during the mirror condition: Bayley in mirror condition (*Mdn* = 81.5), baseline condition (*Mdn* = 0.0) (*U* = 19.5, *p* < .001, η^2^ = .30), and control condition (*Mdn* = 0.0) (*U* = 36.0, *p* = .009, η^2^ = .16); Foster in mirror condition (*Mdn* = 427), baseline condition (*Mdn* = 0.0) (*U* = 3.0, *p* < .001, η^2^ = .40) and control condition (*Mdn* = 25.5) (*U* = 2.0, *p* < .001, η^2^ = .36). No significant difference was found between durations for baseline and control conditions for either dolphin: Bayley (*U* = 18.0, *p* = .60, η^2^ = .02); Foster (*U* = 7.5, *p* = .05, η^2^ = .32).

A total of 12 mark tests were conducted (Foster *n* = 9, Bayley *n* = 3). Notably, both dolphins passed their first mark test; Bayley passed 1 out of 3 mark tests and Foster passed 5 out of 9 mark tests. In all positive mark tests, the dolphins oriented the marked area of their body to the mirror and did so more often in the post-mark than the pre-mark condition (S3 Video, sequence 1 & 2; S4 Video, sequence 1). Mark tests were scored as ambiguous if the dolphins oriented the marked part of their body to the mirror in the post-mark condition, but not significantly more than in the pre-mark condition. In 3 of the 9 mark tests conducted with Foster, the marker malfunctioned resulting in a partially visible or non-visible mark. We considered these tests comparable to the late sham mark tests reported in Reiss & Marino [7]. In the latter and in our late sham-like tests, the dolphins had prior experience being marked and passed at least one mark test. In both studies, the dolphins oriented to the sham-marked area. In two of these late sham-like conditions, Foster oriented more to the area sham-marked in the post-mark condition than the pre-mark condition; therefore, these sham-like tests were considered passing mark tests. Bayley and Foster each failed 1 mark test because they did not approach the mirror in the post-mark condition and this was scored as *not at mirror* (Table 2).

**Table 2:** Date of mark test sessions, age at mark test, location of mark, and mark test results for each dolphin.

Dolphin	Session Date	Age		Location of Mark	Mark Test Results
		Yrs	Mos		
Bayley					
	02/25/11	2	6.5	Mark between pectoral fins	Passed
	11/01/11	3	3	Mark right side of head	Ambiguous
	12/14/11	3	4.5	Mark between pectoral fins	Not at mirror
Foster					
	11/13/09	2	2	Mark left side of head	Passed
	12/03/09	2	3	Mark between pectoral fins*	Ambiguous
	01/06/10	2	4	Mark between pectoral fins	Ambiguous
	01/07/10	2	4	Mark right side of head	Not passed
	01/22/10	2	4.5	Mark left side of head	Not at mirror
	06/16/10	2	9	Mark between pectoral fins*	Passed
	06/17/10	2	9	Mark palmar side of pectoral fins*	Passed
	06/25/10	2	9.5	Mark between pectoral fins	Passed
	11/16/11	4	2	Mark between pectoral fins	Passed

The * indicates sessions when there was a malfunction with the marker and the mark was only partially visible or not visible.

Bayley passed the first mark test conducted when she was 2 years, 7 months of age. After being marked between her pectoral fins, she exhibited ventral orientations at the mirror with repetitive head and body movements in the vertical plane and she repeatedly stretched her neck upward (S4 Video, sequence 2). These behaviors were observed only in the post-mark condition of this session and were not observed in the pre-mark condition. Foster passed the first mark test conducted when he was 2 years, 2 months of age. In the post-mark condition, he repeatedly tilted his head and body to the right, thus exposing the marked area of his head to more light from above, which appeared to make the mark more visible in the mirror. During Foster’s 9^th^ and final mark test, which he passed, he was marked between his pectoral fins and appeared highly motivated to investigate the mark. In the post-mark condition, he swam directly to the mirror within 5 seconds after being released by the trainers post-marking and repeatedly oriented his ventral surface to the mirror, arched and tilted his body so that he was almost inverted, and lifted his pectoral fins exposing the mark to the mirror (S3 Video, sequence 3).

## Discussion

Our findings provide the first comparative ontogenetic data on the emergence of MSR in dolphins. Our results indicate that self-directed behavior at a mirror emerged in a dolphin at 7 months of age, much earlier than has been reported for humans and chimpanzees. Both dolphins exhibited predominantly self-directed behavior at the mirror after minimal mirror exposure and both passed subsequent mark tests. Due to husbandry concerns regarding the marking of young dolphins, we were unable to conduct mark tests before they were two years of age, the time of onset of MSR in children. In this study we considered the dolphins’ performance of self-directed behavior at the mirror the primary evidence for MSR and their subsequent passing of mark tests, when we were able to conduct them, as further confirmation of this ability. Therefore, the age at which they passed the mark tests should not be considered indicative of when MSR emerges in this species. We suggest that the performance of mirror mediated self-directed behaviors is a better behavioral indicator of this ability. Gallup [30] underscored the importance of self-directed behavior as a valid indicator of MSR:

I have never maintained that the mark test is the sine qua non of self-recognition. Appropriate behavior in response to unobtrusively applied facial marks that can only be seen in a mirror constitutes a means of validating impressions that arise out of seeing animals use mirrors in ways that suggest they realize that their behavior is the source of the behavior depicted in the reflection. In trying to demonstrate self-recognition in other species, some people appear to have lost sight of this and have focused almost exclusively on the mark test (p. 42).

Both dolphins produced a rich suite of self-directed behaviors at the mirror. Many of these were combinations of multiple behaviors, such as, open mouth viewing and vertical head movements all while inverted. On several occasions, they also produced instances of what appeared to be “apparent social behavior” [4] and other creative bubble production and play behaviors. Curiously, in certain later sessions, long after the dolphins showed compelling evidence of predominantly mirror self-directed behavior, they performed occasional events of what appeared to be social displays imbedded within sequences of otherwise self-directed behaviors (Fig 1A and 1B). We suggest that these unusual and rare occurrences of social displays were self-directed in nature and examples of solitary play. Similar observations of “apparent social behaviors” by self-recognizing chimpanzees at mirrors have been interpreted as the chimpanzees monitoring their social displays [4]. In wild dolphin calves, as young as 3–4 weeks of age, solitary play involving social behaviors (e.g. head jerks & jaw claps) has been observed [23]. According to Mann and Smuts [23], these social behaviors were considered solitary play because they were exhibited when the calves were alone (< 10m from others). Consistent with these speculations, in sessions 14 and 15, Foster spent a substantial amount of time at the mirror showing predominantly self-directed behavior. Yet in these sessions, during bouts of sociosexual interactions with conspecifics, Foster departed from these interactions, stationed at the mirror, and displayed ‘apparent social behaviors’. We suggest that the dolphins in this study began to use the mirror as a tool to view themselves as they performed a variety of novel and sometimes ‘apparent social behaviors’.

The dolphins also produced several types of novel bubble behaviors and engaged in bubble play at the mirror; these behaviors were not observed in the absence of the mirror in baseline and control conditions. Several studies have previously described and quantified dolphin bubble production and play behavior in the wild and captivity [31–34]. One of the most compelling mirror-mediated bubble behavior events we observed was inverted bubble production and play. During this behavior, the male dolphin (Foster) produced a bubble burst or multiple bubbles and proceeded to smash the bubbles with his head or rostrum (sometimes biting the bubbles) creating a cloud of bubbles, all while in an inverted position (S5 Video). We suggest that much of the bubble behavior performed at the mirror is self-directed solitary play.

Notably, both dolphins passed their first mark tests. In some of the subsequent mark tests, classified as ambiguous, the dolphins oriented to the mark when at the mirror, but failed to show a sufficiently differential behavioral response in the post-mark and pre-marked conditions. Prior MSR studies with chimpanzees and Asian elephants [8] have reported a decline in interest in the mark during a mark test session and after initial mark tests. These responses have been attributed to a possible loss of mark salience over time or to learning the mark is inconsequential [12,30]. This phenomenon has also been observed in MSR studies with children. A longitudinal study with children between the ages of 12 and 24 months, demonstrated that one child who was confirmed to exhibit MSR at 18 and 21 months failed to show MSR at 24 months [14].

The inherent challenges of life in a marine environment have led to the selection of advanced locomotor muscle, sensorimotor and proprioceptive development [20,25,21] in young dolphins. The infant dolphin’s early life within a complex fission-fusion social structure likely contributes to the early development of social awareness [22–24]. This report demonstrates that self-awareness in dolphins co-emerges early in development with precocious social awareness and advanced sensorimotor skills. This may account for the precocious emergence of self-awareness in these dolphins as compared to children and chimpanzees. In dolphins, as in children, these capacities appear to be antecedent or contributing factors in the unfolding of self-awareness. The elucidation of sociocognitive and sensorimotor factors that provide a substrate for self-awareness in dolphins, another large-brained highly social mammal, is critical to our understanding of the evolution of intelligence in the animal world. Future developmental MSR studies with dolphins should expose calves to mirrors from birth to determine the earliest age of onset of self-directed and mark-directed behavior. Observing the behavioral reactions of young dolphins to mirrors provides us with a window into their early cognitive development and the emergence of an awareness of self.

## Supporting information

S1 Table Bottlenose dolphin mirror self-recognition (MSR) ethogram and categorization of behaviors. (DOCX)

S2 Table Age range during mirror exposure, number of sessions, and total mirror exposure time for each dolphin. (DOCX)

S1 Video A compilation of Bayley exhibiting self-directed behavior at the mirror (sequence 1: age 7 months; sequence 2: age 10 months; sequence 3: age 2 years, 2.5 months). The repetitive behavior in sequence 2 was conservatively categorized as contingency testing, although it was exhibited concurrent with self-directed behavior.
(MP4)

S2 Video A compilation of Foster exhibiting self-directed behavior at the mirror (sequence 1: age 14 months; sequence 2: age 16.5 months; sequence 3: age 21 months). The bubbles and bubble streams emitted in sequence 3 were produced in the absence of vocalizations.
(MP4)

S3 Video Sequence 1 (pre-mark) and sequence 2 (post-mark) show Foster’s behavior at the mirror at age 2 years, 9 months during his 7^th^ mark test session. In sequence 2 he is marked on the palmar side of both pectoral fins, but the mark is not easily visible. These two sequences show Foster’s differential response in the 2 conditions. The post-mark condition shows Foster continuously positioning his body to expose the marked area to the mirror. In sequences 1 and 2 the bubble behavior was produced in the absence of vocalizations. Sequence 3 shows Foster at age 4 years, 2 months during his 9^th^ mark test session positioning his body to expose the mark between his pectoral fins.
(MP4)

S4 Video Sequence 1 (pre-mark) and sequence 2 (post-mark) show Bayley’s behavior at the mirror at age 2 years, 6.5 months during her 1^st^ mark test session. In the post-mark condition, she was marked between her pectoral fins with a black triangle and when she initially approached the mirror the mark was intact. In sequence 2 (post-mark condition), upon returning to the mirror the mark was smudged and thus appears larger. In sequence 2 Bayley is continuously positioning her body to expose the marked area to the mirror. These two sequences show Bayley’s differential response in the 2 conditions.
(MP4)

S5 Video Foster (age 21 months) exhibiting inverted bubble production and bubble play at the mirror, an example of self-directed behavior. Foster is producing bubble bursts and multiple smaller bubbles. In this sequence, he bites the bubbles or smashes the bubbles with his head or rostrum creating a cloud of bubbles, while in an inverted position.
(MP4)
