# Supplementary material for: Precocious development of self-awareness in dolphins
Source: PLoS One. 2018 Jan 10;13(1):e0189813. doi: 10.1371/journal.pone.0189813 (PMC5761843; doi:10.1371/journal.pone.0189813)
Supplement: S1 Table — (DOCX) [file pone.0189813.s001.docx]

S1 Table. Bottlenose Dolphin Mirror Self-recognition (MSR) Ethogram and Categorization of Behaviors.

| Categories | Behavior (Code) | Description |
| --- | --- | --- |
| ***Self-directed*** | |  |
| SD | Arch (AR) | Flexing body backwards so head and tail position body into a “u” shape. |
| SD | Airplane (AP) | Pectoral fins positioned perpendicular to body. Head may be stretched up or head-on. |
| SD | Blowhole Kiss (BK) | While performing a head butt on mirror, blowhole makes contact with mirror and is opened and closed producing suction and a “kissing” sound. |
| SD | Bubble Production (BP) | Any bubbles that are produced from blowhole or mouth. |
| SD | Barrel Roll (BR) | In horizontal position body is rotated continuously 360˚. Can be once or repetitive. |
| SD | Body Splay Window (BSW) | Swim up to mirror and in one continuous motion slide ventral surface of body across the window with pectoral fins perpendicular to body and neck stretched up. Can be once or repetitive from any direction. |
| SD | Close Eye (CE) | One eye (either left or right) is positioned close to the mirror. |
| SD | Corkscrew (CS) | Barrel roll in the vertical plane. Can be once or repetitive. |
| SD | Head Rotation (HR) | Circular movement of only head, clockwise or counterclockwise. Can be once or repetitive. |
| SD | Head Stand (HS) | Dorsal side of body facing mirror, positioned vertically with head oriented at the bottom of the mirror. |
| SD | Head Tilt (HT) | Body vertical with small movement of head to left or right. Can be once or repetitive. |
| SD | Horizontal Body Tilt (HBT) | In horizontal position body is rotated 90˚ to 180˚. |
| SD | Horizontal Head Movement (HHM) | Head moved in horizontal plane from left to right. Can be once or repetitive. |
| SD | Near and Far (NF) | Swim up to mirror and then away from mirror in same plane. Also used when dolphin pushes off the mirror with head and then floats away. |
| SD | Neck Stretch (NS) | Extension of neck to move head up or down. Can be once or repetitive. |
| SD | Open Mouth (OM) | Mouth open, can be a small amount or a large amount (wide). |
| SD | Peek-a-boo (PB) | Moving head/eye in and out of mirror frame. Can be once or repetitive. |
| SD | Penis Display (PD) | Protrusion/protraction of penis while at the mirror. |
| SD | Pec Shimmy (PS) | Movement of one or both pectoral fins. Body may be positioned in either vertical or horizontal plane. |
| SD | Rocking Horse (RH) | In vertical position, beginning with head, body is rocked forward 90˚ and then backward to starting position. Can be once or repetitive. |
| SD | Somersault (SS) | In vertical or horizontal plane, beginning with head, body is rocked forward and continues 360˚. |
| SD | Stretch Sway (SW) | Body vertical with neck stretched up and moving head from side to side in a swaying motion. |
| SD | Vertical Body Tilt (VBT) | Body oriented vertically and is moved side to side in a pendulum manner. |
| SD | Vertical Head Movement (VHM) | Head moved in vertical plane up and down. Can be once or repetitive. |
| ***Stationing*** |  |  |
| ST | Arrive (A) | Dolphin approaches and remains at mirror for more than 3 seconds. |
| ST | Eye Edge (EE) | One eye visible at the edge of window, while stationary. |
| ST | Head On (HO) | Body oriented perpendicular to mirror with head facing mirror and stationary. |
| ST | Orient (O) | Dolphin is stationary and the head is positioned either to the left (L) or right (R). Dolphins’ body can be vertical or head-on. |
| ST | Station (ST) | Body is positioned at mirror and not moving. Body can be vertical (dorsal or ventral facing mirror) or parallel (only one side of body facing mirror). |
| ST | Short Station (SST) | Brief (<4 sec) stationing at mirror. |
|  |  |  |
| ***Ambiguous*** |  |  |
| AM | Circling (C) | Swimming by window repeatedly and returning to window frame within 3 seconds. |
| AM | Head Butt (HB) | Head is stretched down and melon and blowhole makes contact with window in a forceful manner. Often hear a “thump” when head makes contact with window. Can be once or repetitive. |
| AM | Lateral Body Rub (LBR) | Dolphin rubs left or right side of head and body against window, often accompanied by rostrum contact and often when vertical. |
| AM | Quiver Jerk (QJ) | Sudden jerky movement of entire body. |
| AM | Rostrum Contact (RC) | End of rostrum touches window, often causing a “squeak” sound like a squeegee rubbing the glass. |
| AM | Spy Hopping (SH) | Dolphin’s body is vertical as they move up to surface and back down. Head goes past surface of water. |
| AM | Swim By (SB) | Swim by window slowly (at least 3 seconds or more) often accompanied by dolphin looking into mirror. |
| ***Social*** |  |  |
| SO | Echolocate (E) | Producing clicks at the mirror. |
| SO | Head Jerk (HJ) | Quick jerky movement of head up. |
| SO | Jaw Clap (JC) | Quick forceful closing of lower and upper jaw creating a loud popping sound. |
| SO | Whistle Bubble Stream (WBS) | Bubble stream produced concurrent with whistle production. |
| SO | Yaw (YW) | Production of a squawky vocalization, mouth open or closed. |
| Modifiers (Put before or after behavior codes) | | |
| D | Distant | Dolphin is oriented towards mirror approximately 5 feet away. |
| Dr | Dorsal | Modifier put after behavior code. |
| I | Inverted | Modifier put before behavior code. |
| L | Left | Modifier put before behavior code |
| PI | Partial Inverted | Modifier put before behavior code |
| r | Repetitive | Repeating a single behavior at least twice. Modifier put before behavior code |
| R | Right | Modifier put before behavior code |
| S | Small | Modifier put after open mouth (OMS). |
| V | Ventral | Modifier put after behavior code |
| W | Wide | Modifier put after open mouth (OMW). |

*Note:* To be conservative, repetitive behaviors were categorized as contingency testing, although they may have been self-directed.
