# Supplementary material for: Precocious development of self-awareness in dolphins
Source: PLoS One. 2018 Jan 10;13(1):e0189813. doi: 10.1371/journal.pone.0189813 (PMC5761843; doi:10.1371/journal.pone.0189813)
Supplement: S2 Table — (DOCX) [file pone.0189813.s002.docx]

S2 Table. Age range during mirror exposure, number of sessions, and total mirror exposure time for each dolphin.

| Dolphin |  | Age range | | | Sessions | Total Mirror Exposure Time | |
| --- | --- | --- | --- | --- | --- | --- | --- |
|  |  | Year | Month | Day | *N* = | Hour | Minutes |
| Bayley |  |  |  |  |  |  |  |
|  | Start of study | 00 | 03 | 27 | 36 | 33 | 24 |
|  | End of study | 03 | 04 | 20 |  |  |  |
| Foster |  |  |  |  |  |  |  |
|  | Start of study | 00 | 14 | 13 | 35 | 32 | 07 |
|  | End of study | 04 | 02 | 07 |  |  |  |

*Note:* Total Mirror Exposure Time is the amount of time the mirror was present for each

session, summed over the number of sessions.
